# Risk of Care Home Placement following Acute Hospital Admission: Effects of a Pay-for-Performance Scheme for Dementia

**DOI:** 10.1371/journal.pone.0155850

**Published:** 2016-05-26

**Authors:** Panagiotis Kasteridis, Anne Mason, Maria Goddard, Rowena Jacobs, Rita Santos, Beatriz Rodriguez-Sanchez, Gerard McGonigal

**Affiliations:** 1 Centre for Health Economics, University of York, York, United Kingdom; 2 Department of Economics, Econometrics and Finance, SOM Research Institute, University of Groningen, 9700 AV Groningen, The Netherlands; 3 Department of Medicine for the Elderly, York Teaching Hospital NHS Foundation Trust, York, United Kingdom; Federal University of Rio de Janeiro, BRAZIL

## Abstract

**Introduction:**

The Quality and Outcomes Framework, or QOF, rewards primary care doctors (GPs) in the UK for providing certain types of care. Since 2006, GPs have been paid to identify patients with dementia and to conduct an annual review of their mental and physical health. During the review, the GP also assesses the carer’s support needs, including impact of caring, and ensures that services are co-ordinated across care settings. In principle, this type of care should reduce the risk of admission to long-term residential care directly from an acute hospital ward, a phenomenon considered to be indicative of poor quality care. However, this potential effect has not previously been tested.

**Methods:**

Using English data from 2006/07 to 2010/11, we ran multilevel logit models to assess the impact of the QOF review on the risk of care home placement following emergency admission to acute hospital. Emergency admissions were defined for (a) people with a primary diagnosis of dementia and (b) people with dementia admitted for treatment of an ambulatory care sensitive condition. We adjusted for a wide range of potential confounding factors.

**Results:**

Over the study period, 19% of individuals admitted to hospital with a primary diagnosis of dementia (N = 31,120) were discharged to a care home; of those admitted for an ambulatory care sensitive condition (N = 139,267), the corresponding figure was 14%. Risk factors for subsequent care home placement included older age, female gender, vascular dementia, incontinence, fall, hip fracture, and number of comorbidities. Better performance on the QOF review was associated with a lower risk of care home placement but only when the admission was for an ambulatory care sensitive condition.

**Conclusions:**

The QOF dementia review may help to reduce the risk of long-term care home placement following acute hospital admission.

## Introduction

Dementia is a chronic and progressive condition, characterized by memory loss, mood swings, and difficulties in communication, mobility, reasoning and self-care [1–3]. The disease has a devastating impact on the lives of people living with dementia, their families and carers [4].

In the UK, the number of people living with dementia is predicted to reach two million by 2050, more than doubling the current annual total cost of care [5]. Inpatient care is costly and people with dementia occupy around one in four acute hospital beds [6]. At the end of their hospital stay, patients should not be transferred directly to long-term residential care from an acute hospital ward unless there are exceptional circumstances. Instead, they should be offered care in a non-acute setting, or support packages in their own home [7].

Since 2006, general practitioners (GPs) in England have been paid to identify and annually review their patients with dementia as part of the pay-for-performance scheme known as the Quality and Outcomes Framework (QOF). The QOF dementia review is a face-to-face consultation that addresses the support needs of the patient and their carer, including: the patient’s physical and mental health; the carer’s need for information; the impact of caring on the carer; and communication and coordination arrangements across organisational boundaries [8].

In principle, the QOF dementia review should help facilitate appropriate discharge arrangements and ensure carers are well supported. This has potential to reduce the risk that acute hospital patients with dementia are discharged directly to long-term residential care.

## Methods

We ran multilevel logit models to test the effect of practice performance on the QOF dementia review on the risk of discharge to care home following acute hospital admission. Our response variable was care home placement following emergency hospital admission. As people with dementia often have complex health and social care needs, we adjusted for an array of potential confounders.

### Study sample

We used data from Hospital Episode Statistics (HES) for England and our unit of analysis was a ‘spell’ (or admission) which is defined as the period from hospital admission to hospital discharge. A patient can experience several hospital admissions within the same year. We investigated two types of patients: (1) people admitted to hospital with a primary diagnosis of dementia and (2) people with dementia who were admitted to hospital for treatment of an ambulatory care sensitive condition [9].

We mapped Read codes—the clinical codes used in UK primary care—for the QOF dementia register to the ICD10 diagnostic codes used for hospital care, so that the criteria for identifying hospital admissions matched the eligibility criteria for the QOF dementia register. The final set of ICD10 codes for dementia is provided in Table 1.

**Table 1:** Diagnostic codes for dementia (ICD-10).

ICD-10 code	Disease
F00	Dementia in Alzheimer disease
F01	Vascular dementia
F02	Dementia in other diseases classified elsewhere
F03	Unspecified dementia
G301	Alzheimer disease with early onset
G302	Alzheimer disease with late onset
G308	Other Alzheimer disease
G309	Alzheimer disease, unspecified
G310	Circumscribed brain atrophy (i.e. Frontotemporal dementia (FTD), Pick disease, Progressive isolated aphasia)
G311	Senile degeneration of brain, not elsewhere classified
G318	Other specified degenerative diseases of nervous system
F051	Delirium superimposed on dementia
F107	Mental and behavioural disorders due to use of alcohol: Residual and late-onset psychotic disorder

Source: International Statistical Classification of Diseases and Related Health Problems 10th Revision: http://apps.who.int/classifications/icd10/browse/2016/en

Next, we identified two groups of patients from HES data. The first group of patients (sample 1) had to have one of these dementia codes as a primary diagnosis. The second group (sample 2) included people with dementia who had been admitted to hospital for treatment of an ambulatory care sensitive condition. In sample 2, individuals had to have one of the dementia ICD10 codes coded in either this admission (as a secondary diagnosis) or in a previous hospital admission (as a primary or secondary diagnosis). We restricted the sample of hospitals to acute providers.

We included admissions between 2006/07 and 2010/11 and used 2011/12 data to obtain complete records for unfinished admissions beginning before the end of the financial year (i.e. March 2011). We excluded admissions longer than 270 days as these patients are less likely to have a QOF dementia review during the year. We also excluded elective admissions; admissions in which the patient died in hospital; and admissions from care homes.

### Quality of care

The primary explanatory variable of interest was the quality of care provided by the practice, proxied by the QOF indicator scores for the annual dementia review. The face-to-face review includes four elements: 1) physical and mental health of the patient; 2) the carer’s need for information; 3) impact of caring on carer; and 4) communication and coordination measures with secondary care, and other sectors where relevant. In principle, the annual review should ensure that appropriate support arrangements are in place so that if patients are admitted to an acute hospital ward, then there is less risk that they will be discharged to long-term residential care. The review also offers the opportunity to discuss decisions about long-term care in a proactive and timely manner.

During our study period, GP practices could earn up to 15 points for reviewing their patients with dementia. The exact number of points earned depends on the percentage of patients reviewed and the ‘thresholds’ defined for the QOF indicator—in this case, the lower and upper thresholds were 25% and 60% respectively (achievement could range from 0% to 100%). Practices reviewing fewer than 25% of eligible patients received no points, and those reviewing 60% or more of patients achieved the full 15 points. Practices achieving a rate between these lower and upper thresholds received a proportion of the points. Therefore, a higher QOF review score indicates better performance. The financial reward is based on the number of points and the price per point (about £125 at the time of the study), and is adjusted for practice list size and disease prevalence within the practice [10].

The QOF indicators for dementia were introduced in April 2006, and we compiled panel data covering the financial years 2006/07 to 2010/11. QOF indicator scores are freely available at practice-level (http://qof.hscic.gov.uk/), but are not published at patient-level. GPs may ‘exception report’ individuals considered unsuitable for treatment, or who are newly registered with the practice or newly diagnosed, or who make an informed dissent. We derived two measures of care quality based on the way ‘exception reported’ patients are modelled (see supporting information, S1 Appendix).

### Covariates

Dementia is known to be independently associated with a higher risk of care home placement [11, 12] but other factors are also important. Details of our literature review of predictors of care home placement amongst older individuals (65+) with dementia are available in S2 Appendix.

We developed an evidence-based list of variables to control for confounding influences. We classified these using the behavioural model of health services use: (a) users’ predisposing characteristics, e.g. age, gender; (b) enabling variables, e.g. income, access to services; and (c) need variables, e.g. illness, symptoms, pain [13].

Clinical and (most) demographic characteristics were derived from HES data. For the analysis of admissions for ambulatory care sensitive conditions, we generated dummy variables to distinguish acute, chronic and vaccine-preventable conditions [9].

Informal care is an important predictor of care home placement, but HES does not record whether or not an individual has a carer. We therefore used small area level (Lower Super Output Area or ‘LSOA’) data from the 2001 Census to derive three measures of informal care reflecting different caregiving intensity, and another variable to model the probability that the person lived alone (S1 Appendix provides details of how the measures were derived).

To control for deprivation, we used LSOA data on the uptake of Pension Credit from the Department for Work and Pensions. Pension Credit is a benefit for older people on low incomes and has two parts: guarantee credit, which tops up income; and savings credit, which is available only to people who have saved towards their retirement. Individuals may receive guarantee credit, savings credit, or both. The poorest individuals are likely to receive guarantee credit only.

We used Rural and Urban classifications to identify whether patients resided in urban areas (i.e. settlements with over 10,000 people). To proxy the availability of care home beds with a local area, we calculated the number of care home beds within 10km of each LSOA centroid in each year of our study. We translated these values into a rate (beds per person) using the LSOA population aged 60 and over. S1 Appendix provides further details of how this measure was calculated.

### Model and statistical approach

The dependent variable was a binary variable that took the value of 1 if a person with dementia was discharged to a care home after acute hospital admission. The discharge destination field in HES does not consistently distinguish residential and nursing home care. We therefore defined ‘care home’ to encompass all types of group home care.

As our dependent variable was binary, we used logit models for the analysis. These are estimated by the maximum likelihood method providing estimates that are consistent and asymptotically normal and efficient. Estimated coefficients capture the effects on the log-odds-ratio [14]. For both study samples, we ran five models: a base case analysis [M1] and four sensitivity analyses, to explore local health authority effects [M2], hospital effects [M3], multiple admissions for individual patients [M4] and an alternative specification of the QOF dementia review measure [M5]. S1 Appendix provides technical details of the statistical analyses, and S3 Appendix provides results tables with the marginal effects.

### Data quality and availability

This was a retrospective longitudinal observational study based on routine datasets, and the quality of these datasets is important for ensuring findings are robust. We combined several national data sets, most of which can be accessed without restriction (Table 2). S1 Appendix provides additional information on how the variables were derived.

**Table 2:** Data sources used to generate variables for the analyses.

Data source	Restrictions	Website	Variables derived
Hospital Episode Statistics (HES)	Yes	http://www.hscic.gov.uk/hes	Clinical / demographic characteristics
			Discharge destination
QOF data	No	http://qof.hscic.gov.uk/	QOF dementia review
Office for national Statistics (ONS)	No	http://www.neighbourhood.statistics.gov.uk	Unpaid care
			Living alone
			Urban residential area (population>10k)
Office for national Statistics (ONS)	No	https://www.ons.gov.uk/peoplepopulationandcommunity/populationandmigration/populationestimates	Population estimates used for measures of care home supply.
Department for Work and Pensions (DWP)	No	http://tabulation-tool.dwp.gov.uk/NESS/BEN/pc.htm	Deprivation (Pension Credit claimants)
Care Quality Commission (CQC)	No	http://www.cqc.org.uk/cqcdata	Care home beds: beds per 100 population aged 60+within 10km of LSOA centroid.

Our outcome variable came from the Hospital Episode Statistics (HES). HES contains details of all admissions, outpatient appointments and A&E attendances for all NHS patients treated in NHS and non-NHS hospitals in England. It is a records-based system, with data collected during the patient’s time at hospital. The release of HES data is subject to strict data governance requirements (see Ethics statement below). Prior to release, HSCIC cleans the data and removes duplicates; we also ran in-house checks to identify any remaining data errors.

Our key explanatory variable was the QOF dementia review. QOF data are extracted automatically from GP clinical systems at the end of March each year, and, following verification checks, are used to calculate GP payments [15]. QOF data are freely available and can be merged with HES data using the GP practice code.

The Office for National Statistics (ONS) publishes a wide range of official statistics, including measures of unpaid care and living arrangements that are collected as part of the Census. Measures of population concentration (rurality) are also available from the ONS. These data are freely available (Table 2) and can be merged with HES data using the small area code.

Our measures of deprivation were based on Pensions Credit data. These data, covering all claimants in England, are reported monthly by the Department for Work and Pensions, and can also be merged with HES using the small area code. Finally, our variable capturing the number of care home beds per 100 individuals aged 60 and over combined bed data provided by the Care Quality Commission (CQC) with population data from the ONS. The CQC directories contain a complete list of every care home in England and are updated weekly.

### Ethics statement

This was a retrospective analysis of previously collected, non-identifiable information, and involved no change in the management of patients. Obtaining individual consent was not feasible so patient records were anonymized and de-identified prior to analysis. The Health and Social Care Information Centre (HSCIC) handles requests for de-identified data and has a statutory responsibility to ensure there is an appropriate legal basis to permit the release and subsequent processing of data, that all necessary approvals are in place, and that organisations have appropriate arrangements and safeguards for secure data handling. The HSCIC approved the release of the Hospital Episode Statistics (http://www.hscic.gov.uk/hes) data to the University of York (Data Re-Use Agreements RU115; RU536; RU750).

## Results

**Fig 1:**
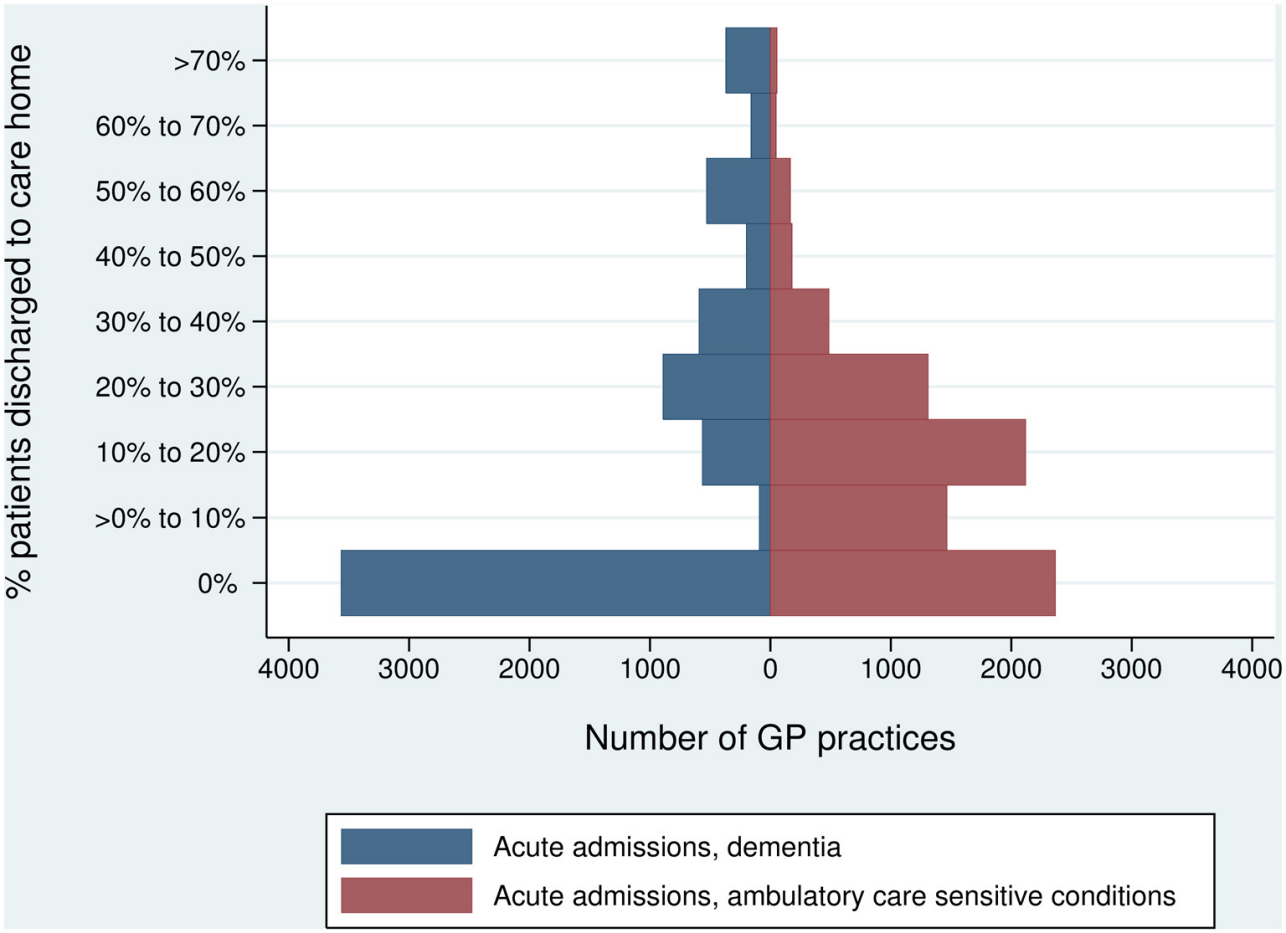
Percentage of acute hospital inpatients discharged to a care home: variation across practices.

From 2006/07 to 2010/11, 31,120 individuals were admitted to hospital with a primary diagnosis of dementia, of which 19% were discharged to a care home (Table 3). For those admitted with ambulatory care sensitive conditions (N = 139,267), the corresponding figure was 14%. There was considerable variation across GP practices with respect to the percentage of their patients who were discharged to long-term care following an acute hospital admission (Fig 1).

**Table 3:** Descriptive statistics for the base case regressions.

Variable	Admissions for dementia (N = 31,120)		Admissions for ACSC (N = 139,267)	
	Discharged to CH	Not discharged to CH	Discharged to CH	Not discharged to CH
N	5,914	25,206	19,694	119,573
Care quality				
QOF dementia score (%)	73.77	73.63	73.44	73.70
Predisposing factors				
Age	83.58	82.08	84.46	82.71
Male	0.35	0.39	0.32	0.36
White ethnicity	0.87	0.86	0.90	0.88
Need factors ^a^				
Alzheimer’s disease	0.25	0.23	0.18	0.18
Vascular dementia	0.30	0.26	0.19	0.17
Urinary incontinence	0.08	0.05	0.07	0.05
Faecal incontinence	0.05	0.03	0.04	0.03
Fall (excludes hip fracture cases)	0.12	0.08	0.11	0.07
Hip fracture (excludes falls)	0.01	0.004	0.02	0.01
Cancer	0.03	0.02	0.03	0.03
Myocardial infarction	0.02	0.01	0.03	0.02
Peripheral vascular disease	0.02	0.02	0.03	0.03
Cerebrovascular disease	0.16	0.13	0.14	0.10
Delirium	0.06	0.05	0.01	0.01
Senility	0.12	0.08	0.10	0.07
Total diagnoses (count) ^b^	5.64	4.70	7.31	6.21
ACSC: Acute ^c^			0.60	0.56
ACSC: Chronic ^d^			0.21	0.29
ACSC: Vaccine ^e^			0.19	0.15
Enabling factors				
Carer 1: % LSOA pop. providing 1–19 hrs/wk unpaid care	6.97	6.90	6.84	6.87
Carer 2: % LSOA pop providing 20–49 hrs/wk unpaid care	1.09	1.11	1.11	1.12
Carer 3: % LSOA pop providing 50+ hrs/wk unpaid care	2.11	2.14	2.11	2.14
Living alone: % LSOA pop aged 60+ living alone	7.29	7.19	7.19	7.13
Deprivation 1: % LSOA pop 60+ claiming guarantee credit	9.47	10.00	9.97	10.11
Deprivation 2: % LSOA pop 60+ claiming savings credit	5.42	5.33	5.62	5.48
Deprivation 3: % LSOA pop 60+ claiming both types of pension credit	11.67	11.50	12.33	11.88
Care home beds: beds per 100 pop 60+within 10km of LSOA centroid	4.85	4.84	4.86	4.88
Urban residential area (population>10k)	0.84	0.83	0.86	0.84
Other				
Year = 2006/07	0.19	0.18	0.17	0.16
Year = 2007/08	0.19	0.18	0.18	0.17
Year = 2008/09	0.20	0.19	0.21	0.20
Year = 2009/10	0.22	0.21	0.22	0.23
Year = 20010/11	0.21	0.23	0.23	0.25

Abbreviations: ACSC: ambulatory care sensitive condition; CH: care home; DWP: Department for Work and Pensions; HES: hospital episode statistics; HF: heart failure; LSOA: Lower Super Output Area; Pop: population; QOF: Quality and Outcomes Framework; MI: myocardial infarction; ONS: Office for National Statistics. ACS conditions based on Bardsley 2013 [9].

^a^ All conditions defined using ICD-10 diagnostic codes.

^b^ Excludes those captured in other covariates.

^c^ Acute: cellulitis, dehydration, dental conditions, ENT infections, gangrene, gastroenteritis, nutritional deficiencies, pelvic inflammatory disease, ulcer, urinary tract infection/ pyelonephritis.

^d^ Chronic: angina, asthma, chronic obstructive pulmonary disease, congestive heart failure, diabetes, epilepsy / convulsions, hypertension, iron deficiency anaemia.

^e^ Vaccine-preventable: influenza, pneumonia, tuberculosis, chronic hepatitis B, other vaccine preventable.

Table 4 shows results from the base case analyses.

**Table 4:** Odds ratios and confidence intervals for dementia and ACS conditions.

	Admissions for dementia		Admissions for ACSC	
	(N = 31,120)		(N = 139,267)	
	OR	95% CI	OR	95% CI
Key explanatory variable				
QOF achievement rate	1.001	0.998, 1.003	0.998***	0.997, 1.000 ^a^
Predisposing factors				
Age	1.022***	1.018, 1.026	1.025***	1.023, 1.027
Male	0.857***	0.803, 0.914	0.875***	0.844, 0.906
White	0.992	0.906, 1.086	1.094***	1.038, 1.154
Need factors				
Alzheimer’s disease	1.191***	1.106, 1.283	1.040*	0.996, 1.085
Vascular dementia	1.192***	1.110, 1.281	1.097***	1.051, 1.145
Urinary incontinence	1.241***	1.070, 1.440	1.230***	1.132, 1.338
Faecal incontinence	1.276**	1.045, 1.558	1.324***	1.188, 1.475
Fall	1.164***	1.046, 1.296	1.196***	1.126, 1.270
Hip fracture	1.477**	1.004, 2.174	1.443***	1.281, 1.625
Cancer	1.380***	1.152, 1.654	1.054	0.962, 1.154
Myocardial infarction	0.985	0.763, 1.270	1.106*	0.997, 1.226
Peripheral vascular disease	0.849	0.681, 1.058	0.867***	0.789, 0.952
Cerebrovascular disease	1.103**	1.010, 1.203	1.253***	1.193, 1.316
Delirium	1.044	0.914, 1.193	1.215**	1.044, 1.415
Senility	1.298***	1.165, 1.446	1.203***	1.130, 1.282
Total diagnoses	1.125***	1.113, 1.138	1.108***	1.103, 1.114
ACSC: Acute (reference)				
ACSC: Chronic			0.710***	0.682, 0.739
ACSC: Vaccine			1.090***	1.043, 1.139
Enabling factors				
% carers 1 to 19 h/w	1.035***	1.010, 1.060	1.015**	1.002, 1.029
% carers 20 to 49 h/w	0.915*	0.835, 1.002	0.955*	0.909, 1.004
% carers > = 50 h/w	0.933***	0.888, 0.981	0.902***	0.877, 0.928
% pop 60+ living alone	0.996	0.987, 1.006	0.990***	0.985, 0.996
% guarantee credit	0.989***	0.984, 0.994	0.988***	0.985, 0.991
% saving credit	1.019**	1.003, 1.036	1.016***	1.007, 1.025
% guarantee & saving credit	1.018***	1.010, 1.027	1.025***	1.020, 1.030
CH Beds/100 pop 60+	1.011	0.987, 1.036	1.000	0.984, 1.016
Urban residential area	1.062	0.970, 1.164	1.097***	1.038, 1.161
Other				
Year = 2007/08	0.953	0.864, 1.052	0.954*	0.903, 1.008
Year = 2008/09	0.931	0.844, 1.026	0.908***	0.861, 0.958
Year = 2009/10	0.854***	0.776, 0.940	0.728***	0.690, 0.767
Year = 2010/11	0.668***	0.605, 0.737	0.612***	0.580, 0.646

Abbreviations: ACSC: ambulatory care sensitive condition; CH: care home; CI: confidence interval; h/w: hours per week; pop: population; QOF, Quality and Outcomes Framework; OR, Odds Ratio.

* p< 0.1,

** p < 0.05,

*** p < 0.01.

^a^ Value rounded to 3 decimal places. The upper confidence interval is 0.99953 (P = 0.008).

For admissions where dementia was the primary diagnosis, the dementia QOF review had no significant impact on the likelihood of care home placement, but there was a small negative effect if the admission was for an ambulatory care sensitive condition. This finding was significant at the 5% level across the five models (see S3 Appendix).

For some factors, the association with risk of care home placement was consistent across all 10 models. In terms of predisposing factors, older age and female gender were associated with significantly higher risk. Need factors that predicted care home placement included incontinence, fall, hip fracture, cerebrovascular disease and senility, as well as a measure of the total number of additional comorbidities. Having cancer was associated with a higher likelihood of being discharged to a care home, but only for those where dementia was the primary reason for the hospital admission. Compared with those admitted for an acute ambulatory care sensitive condition, people admitted to hospital for chronic conditions (such as angina) were significantly less likely to be discharged to a care home, whereas those admitted for vaccine preventable conditions (such as pneumonia or influenza) were at higher risk.

The influence of enabling factors was generally consistent across the models. For example, residing in an area where a higher proportion of people provided at least 50 hours of unpaid care per week was associated with significantly lower risk of care home admission. If the patient’s local neighbourhood was characterised by high uptake of a benefit targeted towards those with greatest income deprivation (modelled by the ‘guarantee’ credit component of Pension Credit) then their risk of placement in long-term care was lower. In all models, local provision of care home beds had no effect on the probability of being discharged to a care home. For some enabling factors, such as whether patients lived in urban areas, or whether they came from a neighbourhood where a high proportion of older individuals lived alone, findings were mixed.

The models also included dummies for each study year. Relative to the baseline year (2006/07), the risk of being discharged from hospital to a care home fell over the study period. This may be due to a number of factors, but efforts to improve the co-ordination of dementia care in both the primary and secondary care sectors may play some part over the period [16].

The signs and the statistical significance of the marginal effects (S3 Appendix), which describe how the explanatory variables affect the probability of discharge to a care home, were consistent with those of the odds ratios (Table 4).

## Discussion

Since 2006, GPs in England have been paid to identify their patients with dementia and offer them an annual review. GPs are responsible for assessing the patient’s mental and physical health, co-ordinating their care and determining the support needs of the patient and their carer. Our study, which uses robust multi-level modelling and five years of data covering all inpatients in England, is the first to address this question. The review was associated with a small but statistically significant reduction in the risk of care home placement but only when the admission was for an ambulatory care sensitive condition, rather than for dementia.

Other important predictors of care home placement were older age, female gender, incontinence, cerebrovascular disease, hip fracture, falls, senility, and comorbidity. The QOF dementia review could target these risk factors to mitigate the risk of care home placement. For instance, people with osteoporosis or mobility problems might be referred to falls clinics; the review of physical health could include an assessment of risk of stroke; and people with incontinence could be referred to continence clinics. The study also found that pension-related benefit payments—in particular, ‘guarantee credit’ which is targeted at those with greater levels of income deprivation—was linked to a lower risk of care home placement, though this finding needs to be verified by further research.

There are several important limitations to the analyses. First, although we used an evidence-based list of variables as covariates, some key predictors of nursing home placement could not be captured in our models due to a lack of data. Around 30% of individuals with dementia have behavioural symptoms [17], but hospitals record only a tiny fraction of cases. Furthermore, hospital data do not capture impaired patient functioning, although urinary incontinence may serve as a proxy for frailty [18]. Evidence shows that unpaid care to be an important predictor of care home placement, but this could only be examined at small area level (LSOAs cover around 1500 individuals). Second, individual-level QOF data are not routinely reported. This means that the QOF score represents a probability of having had a review (though for some practices, the probability is 1, i.e. certain). In addition, QOF scores may not always accurately reflect the quality or appropriateness of care received. Third, our measure of care home admission is based on the discharge field in HES. This does not reliably differentiate type of care home (e.g. nursing home, residential home) and a small proportion (< 5%) are likely to be temporary placements [19, 20].

Many of these limitations could be addressed by a linked longitudinal patient-level dataset that enables individuals to be followed across care settings. Individual-level primary care data would provide greater insight into the QOF dementia review, documenting the timing of the review, what care and support were provided to patients and their carers in the review and subsequently (e.g. referral to social services).

## Conclusions

This study is the first to investigate the effect of the annual QOF dementia review on care home placement following acute hospital admission, a marker of poor quality care. We found the review may reduce the probability of institutionalised care in those with dementia, and we also identified factors associated with a higher risk of institutionalisation that could help inform and guide the care and support provided by GP practices.

Since our study, there has been a significant uplift in the number of points, and therefore rewards, associated with the QOF dementia review, signalling a strengthening of the financial incentives associated with providing good quality primary care for those with dementia. Moreover, additional financial incentive schemes for primary care have been introduced to tackle the perceived high levels of ‘underdiagnosis’ of dementia and to encourage GPs to support patients and carers more effectively [21, 22]. It is not clear how long the QOF system will endure in its current or an amended format, but in many healthcare systems the role of financial incentives and pay-for-performance schemes is likely to be reflected in some way in policy and practice, especially in priority areas such as dementia. Therefore, whatever the fate of the QOF, it is clear that GPs will continue to play a pivotal role in the care of people with dementia and their carers.

## Supporting Information

S1 Appendix Technical Details of the Statistical Approach. (DOCX)

S2 Appendix Findings from the Evidence Review. (DOCX)

S3 Appendix Additional Results. (DOCX)
