# Supplementary material for: Risk of Care Home Placement following Acute Hospital Admission: Effects of a Pay-for-Performance Scheme for Dementia
Source: PLoS One. 2016 May 26;11(5):e0155850. doi: 10.1371/journal.pone.0155850 (PMC4882036; doi:10.1371/journal.pone.0155850)
Supplement: S1 Appendix — (DOCX) [file pone.0155850.s001.docx]

# S1 Appendix: Technical Details of the Statistical Approach

## 1.1 Quality of care

The indicators for dementia were introduced in April 2006, and we compiled a set of panel data covering the financial years 2006/7 to 2010/11. QOF indicator scores are freely available at practice-level (<http://qof.hscic.gov.uk/>), but are not published at patient-level. GPs may ‘exception report’ individuals who are considered unsuitable for treatment, or who are newly registered with the practice or newly diagnosed, or who make an informed dissent.

Let D be the number of patients eligible for the review net of exceptions, E the number of people excluded as exceptions, and N the number of people for whom the indicator is achieved. Then, the percentage of patients receiving the intervention (achievement rate) is given by:

$$QOF=\frac{N}{D+E}\times100$$

As a sensitivity analysis, we also tested ‘underlying achievement’ which differs from in that it excludes exception-reported patients from the denominator (see below).

## 1.2 Neighbourhood variables

Owing to a lack of individual-level data, some of the covariates in the model were measured at small area level (Lower Super Output Area or LSOA) and then assigned to the individual on the basis of their residential area. LSOAs have populations of around 1500 individuals and are based on 2001 population Census data.

Our model included three measures of informal care to reflect different caregiving intensity, and a variable to model the probability that the person lived alone. These were taken directly from the 2001 census. Although these data therefore relate to April 2001 and may not reflect the situation in our study period (2006 to 2010), the 2011 census data use new LSOA boundaries that do not map perfectly onto the old LSOA codes provided in HES.

We also used LSOA data on the uptake of pension credit from the Department for Work and Pensions. This is a benefit for people aged 60 and over living on low incomes. Our measures of pension credit were based on DWP data from 2006/7, which we converted to a percentage rate using LSOA population data on persons aged 60 and over.

We used Rural and Urban classifications to identify whether individual patients resided in urban areas (i.e. settlements with over 10,000 people).

To proxy the availability of care home beds with a local area, we calculated the number of care home beds within 10km of each LSOA centroid. We translated this into a rate (beds per person) using the population aged 60 and over.

We also developed a measure of the availability of care home beds within a local area. Data on care home locations and the number of beds were downloaded from the Care Quality Commission (CQC) website. To calculate annual values of the number of care home beds within 10km of each patient’s LSOA, we estimated the distance from each LSOA a to each care home j and then summed for each LSOA a the number of care homes beds within 10 km. We did this for each of the five years of our study. Since the population at risk of dementia is mostly aged 60 and over, we then converted the number of care homes beds within an area into a rate by dividing by the LSOA population aged over 59. Finally, we converted the rate to a percentage to aid interpretation of the coefficients.

## 1.3 Model and dependent variable

The dependent variable was a binary variable that took the value of 1 if a person with dementia was discharged to a care home after hospital admission. The discharge destination field in HES (disdest) does not consistently distinguish residential and nursing home care, nor does it specify whether the admission to a care home is short-term (e.g. respite, rehabilitation) or long-term (i.e. residence). We therefore defined ‘care home’ to encompass all types of group home care.

As our dependent variable was binary, we used logit models. These are estimated by the maximum likelihood method providing estimates that are consistent and asymptotically normal and efficient. In the logit model, estimated coefficients capture the effects on the log-odds-ratio [1]. Therefore, the results are also expressed as marginal effects – the effects of changes in explanatory variables on the predicted probability of being discharged to care home.

Clustered data structures are widely used in medical and social research [2]. Units of observations are usually grouped into distinct clusters as in the present analysis, where patients are nested in GP practices. One approach to evaluate clustered data is multilevel modelling.

Following the convention, we specified a two-level model where patients are the level 1 units and practices are level 2 clusters. This multilevel structure is appropriate when there is variation (heterogeneity) between clusters. To take into account the nature of the dependent variable and the structure of the data, we specified a multilevel binary response model with random intercept. Using a latent-response formulation the model is defined by

$$y_{ij}*=X_{ij}\beta+a_{j}+\varepsilon_{ij},$$

$$y_{ij}=I(y_{ij}^{*}>0)$$

where i is level 1 (patient-spell) and j is level 2 (GP practice), the latent $y_{ij}^{*}$ is assumed to be linearly related to the vector of explanatory variables $X_{ij}$ and the practice-specific random intercept $a_{j}|X_{ij}\sim N(0,\psi)$ according to . The observed $y_{ij}$indicates discharge to care home and is linked to the latent structure by the measurement equation . The random intercepts $a_{j}$ are assumed to be independent across practices. We assume a logistic distribution for the error term $\varepsilon_{ij}$ which is also assumed to be independent across both patients and practices.

At the post-estimation stage, marginal effects are calculated as the effects of discrete (0 to 1) changes in dummy variables, partial change in age, and unit increments in all other variables on the predicted probabilities. We also calculated percentage changes in probabilities. The effects are averaged over all observations (instead of calculating marginal effects at the mean – see Appendix 3).

## 1.2 Sensitivity analyses

For both study samples, we ran five models: a base case analysis [M1] and four sensitivity analyses [M2 – M5].

Appendix Table 1.1: Overview of the 5 models used for analysis of admissions for dementia (sample 1) and ACSC (sample 2)

| **MODEL** | **PCT fixed effects** | **Hospital fixed effects** | **Multiple spells  per patient** | **QOF review: N/(D+E)** | **QOF review: N/D** |
| --- | --- | --- | --- | --- | --- |
| M1 | 🗶 | 🗶 | 🗶 | ✓ | 🗶 |
| M2 | ✓ | 🗶 | 🗶 | ✓ | 🗶 |
| M3 | 🗶 | ✓ | 🗶 | ✓ | 🗶 |
| M4 | 🗶 | 🗶 | ✓ | ✓ | 🗶 |
| M5 | 🗶 | 🗶 | 🗶 | 🗶 | ✓ |

Key: ACSC: ambulatory care sensitive condition; PCT: primary care trust; QOF: quality and outcomes framework; N: numerator for QOF (number of patients reviewed); D: denominator for QOF (number of patients deemed eligible for review); E: number of patients exception-reported for QOF review.

M1 is the base case analysis, M2-M5 are the sensitivity analyses.

Three of the four sensitivity analyses investigated the complex clustering nature of the data.

PCT level policies (e.g. on the supply of intermediate care) may have an impact on the risk of discharge to long-term care [M2]. However, the inclusion of PCT effects in the model along with LSOA level variables raises some concerns. The higher level PCT dummy variables may mask or even wash out the effects that are assessed at local level. It is critical to retain our LSOA level informal care and deprivation variables as they can inform policy deliberations. Therefore our base case model excluded PCT fixed effects.

Another level of influence is the hospital: patients are clustered within GP practices but are also cross-classified within hospitals (i.e. patients from the same GP practice can be treated in different hospitals; patients in the same hospital come from different GP practices). In the absence of information on hospitals’ multidisciplinary discharge teams, we used hospital fixed effects [M3] to capture these influences. However, these variables also risk masking local level effects.

In addition, some patients had multiple admissions to hospital during the study period 2006/7 to 2010/11. In effect, spells are therefore clustered within patients: in sample 1, there were 31,120 patients with a primary diagnosis of dementia who together had 33,429 admissions (spells); the corresponding numbers for sample 2 were 139,267 patients with dementia who had 195,158 admissions for ambulatory care sensitive conditions. The sensitivity analysis M4 included all spells in the analyses.

In our final sensitivity analysis [M5], we used the reported value for QOF achievement (i.e. the one on which reimbursement is based, known as ‘underlying achievement’). This differs from equation (above) in that it excludes exception-reported patients from the denominator.

## Appendix 1: References

[1] Heij C, de Boer P, Franses PH, Kloek T, van Dijk HK. Econometric Methods with Applications in Business and Economics. Oxford; New York: Oxford University Press, 2004.

[2] Jones AM. Applied Econometrics for Health Economists: A Practical Guide. 2nd edition. London: Office of Health Economics, 2005.
