# Supplementary material for: Risk of Care Home Placement following Acute Hospital Admission: Effects of a Pay-for-Performance Scheme for Dementia
Source: PLoS One. 2016 May 26;11(5):e0155850. doi: 10.1371/journal.pone.0155850 (PMC4882036; doi:10.1371/journal.pone.0155850)
Supplement: S2 Appendix — (DOCX) [file pone.0155850.s002.docx]

# S2 Appendix: Findings from the Evidence Review

An overview of the evidence is shown in Appendix Table 2.1. We searched Medline, EMBASE and PsycINFO for relevant primary research, regression analyses and systematic reviews. Studies were excluded if they focussed on individuals who had been previously admitted to a nursing home.

### 2.1 Users’ predisposing characteristics

In most studies, older age was positively associated with risk of institutionalisation [1-11], though many studies found no effect after controlling for other factors [12-19]. The impact of gender was inconsistent, but white ethnicity was positively associated with risk of care home placement in 5 of the 6 studies that assessed its impact [2, 3, 6, 10, 11].

Most studies found that married care recipients were at a lower risk of institutionalisation [3-5, 10, 11, 14, 16, 19, 20]. The impact of having an unpaid carer depended on the relationship with the patient: a spouse carer was associated with lower risk [7, 10, 12, 15, 20-22], whereas the impact of an adult child carer was less clear, with studies reporting positive [10, 12], negative [6, 7] and insignificant [14] effects.

### 2.1 Enabling factors

Neither patient income [11, 13, 14] nor educational level [4, 11, 13-15, 17, 19, 21] exerted a consistent influence on the risk of institutionalisation, but people with dementia were more likely to be institutionalised if their carer had a higher income [10, 20, 22] and/or was employed [10, 12, 20, 23]. The effects of social support services such as adult day care, and home-help varied widely across studies [10].

### 2.3 Need factors

In the behavioural model of health services use, need variables are separated into primary and secondary stressors. Primary stressors include objective patient and carer need, such as type, severity and duration of the person’s illness, including dementia, cognitive and functional impairment and the behavioural and psychological symptoms of dementia. Weekly caregiving hours and objective measures of carer health are examples of primary stressors. Secondary stressors relate to subjective measures such as carer burden and carer quality of life.

#### 2.3.1 Primary stressors

A higher risk of institutionalisation was, unsurprisingly, associated with higher levels of impaired functioning in the activities of daily living (e.g. bathing, dressing or shopping) [3-8, 10, 11, 14, 16, 17, 20, 23, 24]. Sleeping abnormalities [6, 20], anxiety [24] depression [10, 14, 24], delirium [10, 24] and incontinence [17] were also risk factors for long-term care.

Relative to other types of dementia, Alzheimer’s disease was associated with a higher risk of care home placement [7, 10, 14, 19, 21]. Dementia severity [5, 7, 10, 11, 20] was a risk factor for institutionalisation, but prior hospitalization had no significant effect [11].

Different health problems were also evaluated through a wide range of studies: there was some evidence of a heightened risk of care home admission associated with diabetes [3, 11], stroke [3, 19], heart and/or cerebrovascular disease [1], cancer [3] hip fracture [1] and falls [3]. Evidence on the impact of hypertension, arthritis, respiratory disease, and visual or hearing impairment was inconclusive [11]. Caregiver´s health status [7, 10, 23] was strongly associated with higher risk of institutionalization for the person with dementia. Functional dependency [6, 10, 22] and depression [6, 10, 23] in carers were linked to an elevated risk of care home placement.

#### 2.3.2 Secondary stressors

Caregiver self-related quality of life, caregiver subjective burden [6, 7, 20] and stress [12, 14, 22] were potential determinants of a shorter time to care home admission. However, evidence on the impact of caregiving hours was inconsistent.

Appendix Table 2. 1: Overview of the evidence

| **Variable** | **Association with risk of institutionalisation** | **Covariate name** |
| --- | --- | --- |
| ***Patient’s predisposing characteristics*** |  |  |
| Age | Positive [1-11] | age |
|  | Insignificant [12-19] |  |
| Gender (male) | Positive [3, 10] | male |
|  | Negative [1, 20] |  |
|  | Insignificant [2, 4, 9, 11-19] |  |
| Ethnicity (white) | Positive [2, 3, 6, 10, 11] | white |
| Lives alone | Positive [3, 6, 8, 10] | % elderly living alone (LSOA) |
|  | Insignificant [11, 13] | (Lower Super Output Area) |
|  | Inconsistent [14] |  |
| Marital status | Negative [3-5, 10, 11, 14, 16, 19, 20] | Not assessed |
|  | Positive [2] |  |
| Unpaid carer | Negative (spouse) [7, 10, 12, 15, 20-22] | Carer measures 1 – 3 (LSOA) |
|  | Inconsistent (adult child) [6, 7, 10, 12, 14] |  |
| ***Enabling factors*** |  |  |
| Patient income | Positive [3] | Deprivation measures 1-3 (LSOA) |
|  | Insignificant [11, 13, 14] |  |
| Patient education | Negative [4, 10] | Not assessed |
|  | Insignificant [11, 13-15, 17, 19, 21] |  |
| Home ownership | Insignificant [3, 11] | Not assessed |
| Carer income | Positive [10, 20, 22] | Not assessed |
| Carer employment | Positive [10, 12, 20, 23] | Not assessed |
| Adult day care | Inconsistent [10] | Not assessed |
| Home-help | Inconsistent [10] | Not assessed |
| ***Need factors: primary stressors*** |  |  |
| Alzheimer’s disease | Positive [7, 10, 14, 19, 21] | Alzheimer’s disease |
| Anxiety / depression | Positive [10, 14, 24] | Not assessed |
| Arthritis | Inconsistent [11] | Not assessed |
| Cancer | Positive [3] | Cancer |
| Delirium | Positive [10, 24] | Delirium |
|  |  | Senility |
| Dementia severity | Positive [5, 7, 10, 11, 20] | Not assessed |
| Diabetes | Positive [3, 11] | Not assessed |
| Falls | Positive [3] | Fall (excludes hip fracture) |
| Heart disease | Positive [1] | Peripheral vascular disease |
|  |  | Myocardial infarction |
| Hip fracture | Positive [1] | Hip fracture |
| Hypertension | Inconsistent [11] | Not assessed |
| Incontinence | Positive [17] | Urinary incontinence |
|  | Inconsistent [14] | Faecal incontinence |
| Aggressive behaviour | Positive [10] | Not assessed |
|  | Insignificant [21] |  |
| Psychotic symptoms | Positive [6] | Not assessed |
|  | Insignificant [21] |  |
| Patient functioning / Activities of Daily Living | Positive [3-8, 10, 11, 14, 16, 17, 20, 23, 24] | Not assessed |
| Prior hospitalisation | Insignificant [11] | Not assessed |
| Prior nursing home admission | Positive [3] | Not assessed |
| Respiratory disease | Inconsistent [11] | Not assessed |
|  | Negative [2] |  |
| Sleeping abnormalities | Positive [6, 20] | Not assessed |
| Stroke | Positive [1, 3, 19] | Cardiovascular disease |
| Visual / hearing impairment | Inconsistent [11] | Not assessed |
| Carer health status | Positive [7, 10, 23] | Not assessed |
| Carer functional impairment | Positive [6, 10, 22] | Not assessed |
| Carer depression | Positive [6, 10, 23] | Not assessed |
| ***Need factors: secondary stressors*** |  |  |
| Carer quality of life | Positive [6, 7, 20] | Not assessed |
| Carer burden | Positive [6, 7, 20] | Not assessed |
| Carer stress | Positive [12, 14, 22] | Not assessed |
| Carer hours | Inconsistent [6, 13, 20] | Not assessed |

## Appendix 2: References

1. Aguero-Torres H, von Strauss E, Viitanen M, Winblad B, Fratiglioni L. Institutionalization in the elderly: the role of chronic diseases and dementia. Cross-sectional and longitudinal data from a population-based study. J Clin Epidemiol. 2001;54(8):795-801.

2. Andel R, Hyer K, Slack A. Risk factors for nursing home placement in older adults with and without dementia. J Aging Health. 2007;19(2):213-28.

3. Gaugler JE, Duval S, Anderson KA, Kane RL. Predicting nursing home admission in the U.S: a meta-analysis. BMC Geriatr. 2007;7:13. PubMed Central PMCID: PMCPMC1914346

4. Smith GE, Kokmen E, O'Brien PC. Risk factors for nursing home placement in a population-based dementia cohort. J Am Geriatr Soc. 2000;48(5):519-25.

5. Smith GE, O'Brien PC, Ivnik RJ, Kokmen E, Tangalos EG. Prospective analysis of risk factors for nursing home placement of dementia patients. Neurology. 2001;57(8):1467-73.

6. Yaffe K, Fox P, Newcomer R, Sands L, Lindquist K, Dane K, et al. Patient and caregiver characteristics and nursing home placement in patients with dementia. JAMA. 2002;287(16):2090-7.

7. Hebert R, Dubois MF, Wolfson C, Chambers L, Cohen C. Factors associated with long-term institutionalization of older people with dementia: data from the Canadian Study of Health and Aging. J Gerontol A Biol Sci Med Sci. 2001;56(11):M693-9.

8. Drame M, Lang PO, Jolly D, Narbey D, Mahmoudi R, Laniece I, et al. Nursing home admission in elderly subjects with dementia: predictive factors and future challenges. J Am Med Dir Assoc. 2012;13(1):83.e17-20. doi: 10.1016/j.jamda.2011.03.002.

9. Jagger C, Andersen K, Breteler MM, Copeland JR, Helmer C, Baldereschi M, et al. Prognosis with dementia in Europe: A collaborative study of population-based cohorts. Neurologic Diseases in the Elderly Research Group. Neurology. 2000;54(11 Suppl 5):S16-20.

10. Luppa M, Luck T, Brahler E, Konig HH, Riedel-Heller SG. Prediction of institutionalisation in dementia. A systematic review. Dement Geriatr Cogn Disord. 2008;26(1):65-78. doi: <http://dx.doi.org/10.1159/000144027>.

11. Luppa M, Luck T, Weyerer S, Konig HH, Brahler E, Riedel-Heller SG. Prediction of institutionalization in the elderly. A systematic review. Age Ageing. 2010;39(1):31-8. doi: <http://dx.doi.org/10.1093/ageing/afp202>.

12. Colerick EJ, George LK. Predictors of institutionalization among caregivers of patients with Alzheimer's disease. J Am Geriatr Soc. 1986;34(7):493-8.

13. Fisher L, Lieberman MA. A longitudinal study of predictors of nursing home placement for patients with dementia: the contribution of family characteristics. Gerontologist. 1999;39(6):677-86.

14. Gaugler JE, Yu F, Krichbaum K, Wyman JF. Predictors of nursing home admission for persons with dementia. Med Care. 2009;47(2):191-8. doi: 10.1097/MLR.0b013e31818457ce.

15. Lieberman MA, Kramer JH. Factors affecting decisions to institutionalize demented elderly. Gerontologist. 1991;31(3):371-4.

16. Severson MA, Smith GE, Tangalos EG, Petersen RC, Kokmen E, Ivnik RJ, et al. Patterns and predictors of institutionalization in community-based dementia patients. J Am Geriatr Soc. 1994;42(2):181-5.

17. O'Donnell BF, Drachman DA, Barnes HJ, Peterson KE, Swearer JM, Lew RA. Incontinence and Troublesome Behaviors Predict Institutionalization in Dementia. J Geriatr Psychiatry Neurol. 1992;5(1):45-52. doi: 10.1177/002383099200500108.

18. Banerjee S, Murray J, Foley B, Atkins L, Schneider J, Mann A. Predictors of institutionalisation in people with dementia. J Neurol Neurosurg Psychiatry. 2003;74(9):1315-6.

19. Luck T, Luppa M, Weber S, Matschinger H, Glaesmer H, Konig HH, et al. Time until institutionalization in incident dementia cases--results of the Leipzig Longitudinal Study of the Aged (LEILA 75+). Neuroepidemiology. 2008;31(2):100-8. doi: <http://dx.doi.org/10.1159/000146251>.

20. Habermann S, Cooper C, Katona C, Livingston G. Predictors of entering 24-h care for people with Alzheimer's disease: results from the LASER-AD study. Int J Geriatr Psychiatry. 2009;24(11):1291-8. doi: 10.1002/gps.2259.

21. Bakker C, de Vugt ME, van Vliet D, Verhey FR, Pijnenburg YA, Vernooij-Dassen MJ, et al. Predictors of the time to institutionalization in young- versus late-onset dementia: results from the Needs in Young Onset Dementia (NeedYD) study. J Am Med Dir Assoc. 2013;14(4):248-53. doi: 10.1016/j.jamda.2012.09.011.

22. Pot AM, Deeg DJ, Knipscheer CP. Institutionalization of demented elderly: the role of caregiver characteristics. Int J Geriatr Psychiatry. 2001;16(3):273-80.

23. Coehlo DP, Hooker K, Bowman S. Institutional placement of persons with dementia: what predicts occurrence and timing? J Fam Nurs. 2007;13(2):253-77. doi: 10.1177/1074840707300947.

24. Steele C, Rovner B, Chase GA, Folstein M. Psychiatric symptoms and nursing home placement of patients with Alzheimer's disease. Am J Psychiatry. 1990;147(8):1049-51.
