# Supplementary material for: Risk of Care Home Placement following Acute Hospital Admission: Effects of a Pay-for-Performance Scheme for Dementia
Source: PLoS One. 2016 May 26;11(5):e0155850. doi: 10.1371/journal.pone.0155850 (PMC4882036; doi:10.1371/journal.pone.0155850)
Supplement: S3 Appendix — (DOCX) [file pone.0155850.s003.docx]

# S3 Appendix: Additional Results

Appendix Table 3.1 Analyses of admissions with a primary diagnosis of dementia: odds ratios for base and sensitivity analysis models

|  | **M1** | **M2** | **M3** | **M4** | **M5** |
| --- | --- | --- | --- | --- | --- |
|  | (N=31,120) | (N=31,120) | (N=31,120) | (N=33,429) | (31,120) |
| QOF achievement rate | 1.001 | 1.001 | 1.001 | 1.001 | 1.001 |
| *Predisposing factors* |  |  |  |  |  |
| Age | 1.022*** | 1.022*** | 1.022*** | 1.021*** | 1.022*** |
| Male | 0.857*** | 0.861*** | 0.860*** | 0.854*** | 0.857*** |
| White | 0.992 | 0.988 | 0.994 | 1.001 | 0.992 |
| *Need factors* |  |  |  |  |  |
| Alzheimer’s disease | 1.191*** | 1.160*** | 1.180*** | 1.200*** | 1.191*** |
| Vascular | 1.192*** | 1.155*** | 1.181*** | 1.173*** | 1.192*** |
| Urinary incontinence | 1.241*** | 1.214** | 1.229*** | 1.293*** | 1.241*** |
| Faecal incontinence | 1.276** | 1.290** | 1.263** | 1.219** | 1.276** |
| Fall | 1.164*** | 1.177*** | 1.185*** | 1.164*** | 1.164*** |
| Hip fracture | 1.477** | 1.340 | 1.392* | 1.516** | 1.478** |
| Cancer | 1.380*** | 1.359*** | 1.373*** | 1.362*** | 1.380*** |
| Myocardial infarction | 0.985 | 1.000 | 0.993 | 0.974 | 0.985 |
| Peripheral vascular disease | 0.849 | 0.838 | 0.848 | 0.869 | 0.849 |
| Cerebrovascular disease | 1.103** | 1.131*** | 1.120** | 1.111** | 1.103** |
| Delirium | 1.044 | 0.999 | 1.016 | 1.052 | 1.044 |
| Senility | 1.298*** | 1.282*** | 1.237*** | 1.277*** | 1.298*** |
| Total diagnoses | 1.125*** | 1.128*** | 1.130*** | 1.123*** | 1.125*** |
| *Enabling factors* |  |  |  |  |  |
| % carers 1 to 19 h/w | 1.035*** | 1.016 | 1.014 | 1.033*** | 1.035*** |
| % carers 20 to 49 h/w | 0.915* | 0.909** | 0.915* | 0.917* | 0.915* |
| % carers >=50 h/w | 0.933*** | 0.939** | 0.933*** | 0.936*** | 0.933*** |
| % pop 60+ living alone | 0.996 | 0.996 | 0.999 | 0.996 | 0.996 |
| % guarantee credit | 0.989*** | 0.995 | 0.995* | 0.988*** | 0.989*** |
| % saving credit | 1.019** | 1.017** | 1.015* | 1.021*** | 1.019** |
| % guarantee & saving credit | 1.018*** | 1.011** | 1.011** | 1.019*** | 1.018*** |
| CH Beds/100 pop 60+ | 1.011 | 1.012 | 0.993 | 1.008 | 1.011 |
| Urban | 1.062 | 1.046 | 1.070 | 1.069 | 1.063 |
| *Other* |  |  |  |  |  |
| Year=2007/08 | 0.953 | 0.955 | 0.955 | 0.970 | 0.952 |
| Year=2008/09 | 0.931 | 0.931 | 0.926 | 0.944 | 0.930 |
| Year=2009/10 | 0.854*** | 0.846 | 0.833*** | 0.864*** | 0.854*** |
| Year=2010/11 | 0.668*** | 0.656 | 0.662*** | 0.689*** | 0.667*** |

Abbreviations: M1: Model 1; M2: Model 2 (PCT fixed effects included); M3: Model 3 (Hospital fixed effects included); M4: Model 4 (multiple spells included); M5: Model 5 (quality measured by underlying achievement); OR: Odds Ratio; QOF, Quality and Outcomes Framework

* p< 0.1, ** p < 0.05, *** p < 0.01

Appendix Table 3.2 Analyses of admissions for ambulatory care sensitive conditions: odds ratios for base and sensitivity analysis models

|  | **M1** | **M2** | **M3** | **M4** | **M5** |
| --- | --- | --- | --- | --- | --- |
|  | (N=139,267) | (N=139,267) | (N=139,267) | (N=195,158) | (N=139,267) |
| QOF achievement rate | 0.998*** | 0.998** | 0.999** | 0.999** | 0.998** |
| *Predisposing factors* |  |  |  |  |  |
| Age | 1.025*** | 1.025*** | 1.026*** | 1.026*** | 1.025*** |
| Male | 0.875*** | 0.879*** | 0.877*** | 0.879*** | 0.875*** |
| White | 1.094*** | 1.083*** | 1.076*** | 1.107*** | 1.094*** |
| *Need factors* |  |  |  |  |  |
| Alzheimer disease | 1.040* | 1.023 | 1.033 | 1.083*** | 1.040* |
| Vascular | 1.097*** | 1.078*** | 1.092*** | 1.134*** | 1.096*** |
| Urinary incontinence | 1.230*** | 1.248*** | 1.244*** | 1.269*** | 1.231*** |
| Faecal incontinence | 1.324*** | 1.332*** | 1.327*** | 1.326*** | 1.324*** |
| Fall | 1.196*** | 1.203*** | 1.206*** | 1.202*** | 1.196*** |
| Hip fracture | 1.443*** | 1.455*** | 1.467*** | 1.462*** | 1.444*** |
| Cancer | 1.054 | 1.059 | 1.054 | 1.064 | 1.053 |
| Myocardial infarction | 1.106* | 1.104* | 1.092* | 1.075 | 1.106* |
| Peripheral vascular disease | 0.867*** | 0.871*** | 0.869*** | 0.898*** | 0.866*** |
| Cerebrovascular disease | 1.253*** | 1.255*** | 1.257*** | 1.247*** | 1.253*** |
| Delirium | 1.215** | 1.249*** | 1.250*** | 1.231*** | 1.214** |
| Senility | 1.203*** | 1.192*** | 1.167*** | 1.198*** | 1.203*** |
| Total diagnoses | 1.108*** | 1.108*** | 1.108*** | 1.105*** | 1.108*** |
| ACSC: Acute (*reference*) |  |  |  |  |  |
| ACSC: Chronic | 0.710*** | 0.710*** | 0.708*** | 0.715*** | 0.710*** |
| ACSC: Vaccine | 1.090*** | 1.104*** | 1.110*** | 1.114*** | 1.090*** |
| *Enabling factors* |  |  |  |  |  |
| % carers 1 to 19 h/w | 1.015** | 0.997 | 0.992 | 1.015** | 1.015** |
| % carers 20 to 49 h/w | 0.955* | 0.946** | 0.925*** | 0.947** | 0.955* |
| % carers >=50 h/w | 0.902*** | 0.914*** | 0.900*** | 0.895*** | 0.902*** |
| % pop 60+ living alone | 0.990*** | 0.991*** | 0.999 | 0.990*** | 0.990*** |
| % guarantee credit | 0.988*** | 0.991*** | 0.992*** | 0.987*** | 0.988*** |
| % saving credit | 1.016*** | 1.016*** | 1.013*** | 1.018*** | 1.016*** |
| % guarantee & saving credit | 1.025*** | 1.018*** | 1.014*** | 1.025*** | 1.025*** |
| CH Beds/100 pop 60+ | 1.000 | 0.994 | 1.006 | 1.001 | 1.000 |
| Urban | 1.097*** | 1.026 | 1.065** | 1.089*** | 1.097*** |
| *Other* |  |  |  |  |  |
| Year=2007/08 | 0.954* | 0.956 | 0.953* | 0.952* | 0.954* |
| Year=2008/09 | 0.908*** | 0.913*** | 0.915*** | 0.893*** | 0.908*** |
| Year=2009/10 | 0.728*** | 0.734*** | 0.737*** | 0.735*** | 0.727*** |
| Year=2010/11 | 0.612*** | 0.624*** | 0.618*** | 0.616*** | 0.612*** |

Abbreviations: M1: Model 1; M2: Model 2 (PCT fixed effects included); M3: Model 3 (Hospital fixed effects included); M4: Model 4 (multiple spells included); M5: Model 5 (quality measured by underlying achievement); ACSC: Ambulatory care sensitive condition; OR: Odds Ratio; QOF, Quality and Outcomes Framework
* p< 0.1, ** p < 0.05, *** p < 0.01

Appendix Table 3.3 Marginal effects

|  | **Admissions for dementia** | | **Admissions for ACSC** | |
| --- | --- | --- | --- | --- |
| Variable | change in | % change in | change in | % change in |
|  | probability | probability | probability | probability |
| *Changes from 0 to 1* |  |  |  |  |
| Male | -0.022*** | -11.98*** | -0.013*** | -10.77*** |
| White | -0.001 | -0.64 | 0.010*** | 9.43*** |
| Alzheimer’s disease | 0.025*** | 15.38*** | 0.008*** | 7.25*** |
| Vascular dementia | 0.025*** | 15.48*** | 0.013*** | 11.72*** |
| Urinary incontinence | 0.032*** | 19.12*** | 0.026*** | 23.14*** |
| Faecal incontinence | 0.037** | 21.75** | 0.031*** | 27.86*** |
| Fall | 0.022*** | 13.20*** | 0.019*** | 17.51*** |
| Hip fracture | 0.061* | 36.37* | 0.043*** | 38.82*** |
| Cancer | 0.050*** | 29.44*** | 0.006 | 5.65 |
| Myocardial infarction | -0.002 | -1.27 | 0.007 | 6.62 |
| Peripheral vascular disease | -0.022 | -12.77 | -0.010*** | -9.09*** |
| Cerebrovascular disease | 0.014** | 8.32** | 0.023*** | 21.29*** |
| Delirium | 0.006 | 3.62 | 0.022*** | 19.86*** |
| Senility | 0.039*** | 23.46*** | 0.019*** | 17.13*** |
| ACSC: Acute (*reference*) |  |  |  |  |
| ACSC: Chronic |  |  | -0.032*** | -25.85*** |
| ACSC: Vaccine |  |  | 0.011*** | 9.96*** |
| Urban | 0.008 | 5.13 | 0.008*** | 7.83*** |
| Year=2007/08 | -0.007 | -3.90 | -0.005** | -4.27** |
| Year=2008/09 | -0.010 | -5.76 | -0.011*** | -9.54*** |
| Year=2009/10 | -0.022*** | -12.26*** | -0.029*** | -23.95*** |
| Year=2010/11 | -0.053*** | -28.67*** | -0.045*** | -35.07*** |
| *Unit change from x to x+1^&^* |  |  |  |  |
| QOF achievement rate | 0.000 | 0.06 | -0.0001** | -0.11** |
| Total diagnoses | 0.017*** | 10.10*** | 0.010*** | 9.17*** |
| % carers 1 to 19 h/w | 0.005*** | 2.86*** | 0.002** | 1.30 |
| % carers 20 to 49 h/w | -0.012** | -7.12** | -0.005** | -4.71 |
| % carers >=50 h/w | -0.010*** | -5.55*** | -0.011*** | -9.37*** |
| % pop 60+ living alone | -0.001 | -0.30 | -0.001*** | -0.87*** |
| % guarantee credit | -0.002*** | -0.91*** | -0.001*** | -1.14*** |
| % saving credit | 0.003** | 1.60** | 0.002*** | 1.60*** |
| % guarantee & saving credit | 0.003*** | 1.50*** | 0.003*** | 2.18*** |
| CH Beds/100 pop 60+ | 0.002 | 0.90 | 0.0001 | 0.12 |
| *Partial change* |  |  |  |  |
| Age | 0.003*** | 1.77*** | 0.003*** | 2.24*** |

^&^ For percentages, a unit change is equivalent to one percentage point change

ACSC: ambulatory care sensitive condition; CH: care home; QOF: quality and outcomes framework
